# Useful clinical features and hematological parameters for the diagnosis of dengue infection in patients with acute febrile illness: a retrospective study

**DOI:** 10.1186/s12878-018-0116-1

**Published:** 2018-08-29

**Authors:** Juthatip Chaloemwong, Adisak Tantiworawit, Thanawat Rattanathammethee, Sasinee Hantrakool, Chatree Chai-Adisaksopha, Ekarat Rattarittamrong, Lalita Norasetthada

**Affiliations:** Division of Hematology, Department of Internal Medicine, Faculty of Medicine, Chiang Mai University, 110 Intravaroros road, A. Muang, Chiang Mai, 50200 Thailand

**Keywords:** Dengue infection, Diagnosis of dengue, Acute febrile illness, Complete blood count, Hematological parameters

## Abstract

**Background:**

Dengue infection patients are presented with acute febrile illness. Clinical presentations may mimic other infections. The serology for definite diagnosis is costly and inaccessible in many hospitals. We sought to identify the clinical features and hematologic parameters from a complete blood count (CBC) which distinguish dengue infection from other causes.

**Methods:**

This was a retrospective single center study from Chiang Mai University Hospital. All patients who presented with acute fever between September 2013 and July 2015 were included. The diagnosis of dengue infection must be confirmed by serology. The control groups were patients who presented with acute febrile illness without localizing signs. Clinical data and CBC results were reviewed and compared. The Chi-square test was used to compare categorical variables. The CBC parameters were analyzed using the linear mixed model.

**Results:**

One hundred and fifty-four dengue and 146 control patients were included. Headache, nausea, loss of appetite and bleeding diathesis were significantly symptoms in dengue patients (*p* < 0.05). There was some diversity in the the CBC in the dengue patients compared to the control group. Moreover, this study also identified the day of fever which these parameters were statistically significant. The dengue group had higher hemoglobin and hematocrit from day 3 to day 10 (*p* < 0.001), lower white blood cell count from day 1 to day 10 (*p* < 0.001), lower platelet count from day 3 to day 10 (*p* < 0.001), higher monocyte on day 1–4 (*p* < 0.001), higher atypical lymphocyte percentage on day 5–9 (*p* < 0.001) and higher eosinophil percentage on day 9–10 (*p* = 0.001). Furthermore, the neutrophil to lymphocyte percentage ratio of dengue group was > 1 on the first 5 days then reversed on day 6 to Day 9 but in non-dengue group, the ratio was always > 1.

**Conclusion:**

We identified important clinical features and CBC parameters to differentiate dengue patients from other patients who had acute febrile illness from other causes. This identification could be done in local hospitals to give an accurate diagnosis, enabling further investigation to be tailored and treatment commenced earlier.

## Background

Dengue is an infection caused by Dengue virus which transmitted by the bite of an infected mosquito. The disease is found in approximately 50 million people worldwide annually and 2.5 billion in dengue endemic countries [1]. The data from the population of Thailand from 1 January 2016 to 20 November 2016 shows a total of 34,677 cases, a morbidity rate of 0.01/100,000 population [2].

Dengue infection severity varies from mild illness to dengue shock syndrome. The clinical presentation of dengue patients is acute febrile illness with no localizing signs and symptoms which may mimic other infections. Therefore the laboratory tests such as a complete blood count (CBC), serological test or blood culture need to be used to differential and confirm the diagnosis.

The CBC in dengue patients change by the day of the fever, specifically on days 3 to 8, starting with progressive leukopenia followed by thrombocytopenia and hemoconcentration due to plasma leakage [3, 4]. The data from Brazil demonstrated mean white blood cell count (WBC) of dengue infected patients was 4.6 × 10^9^/L with the lowest count of 0.7 × 10^9^/L and the mean platelet count was 26.4 × 10^9^/L with the lowest count registered was less than 1 × 10^9^/L [5].

Currently, the serological test is used to confirm the diagnosis of dengue infection such as the detection of the dengue NS1 antigen (sensitivity 76% and specificity 98%) or the dengue IgM antibody by the ELISA method (sensitivity 90% and specificity 93%) [6]. Nevertheless, these serological tests may be inaccessible in underdeveloped countries or in some small local hospitals, so the clinical clues from the history taking, physical examination and the routine laboratory tests are still important. There was a study in Puerto Rico in 2011 which revealed that the dengue patients had enhanced leukopenia at 87% and a positive tourniquet test in 52% of patients. Hence patients with acute febrile illness with leukopenia and a positive tourniquet test were more likely to be infected with dengue than influenza, leptospirosis and enteroviruses [7].

The CBC parameters such as hemoglobin (Hb), hematocrit (Hct), WBC count, differential percentages of the WBCs and platelet count alter each day of the fever in patients infected with dengue. Little evidence exists to date to identify these daily changes ensuring dengue infection is distinguished from the other causes of acute febrile illness without localizing signs. Our aim was to find the most useful clinical features and CBC parameters which enable dengue to be distinguished from other infections in cases of acute febrile illness patients.

## Methods

We retrospectively reviewed medical records of patients age 15 years or older who presented at Chiang Mai University Hospital for both inpatient and outpatient between September 2013 and July 2015. The inclusion criteria were patients with acute febrile illness (less than 7 days) without any identified source of infection. Patients must have had serology test or blood culture to confirm the diagnosis to be included in the study. Dengue infected patients were identified by a positive result of either the dengue NS1 antigen or dengue IgM antibody. The control group consisted of patients who also presented with fever without localzing signs and symptom including rickettsial infection (scrub typhus IgM or murine typhus IgM positive titer more than 1:400), primary bacteremia (positive blood culture without other primary source of infection), leptospirosis (positive leptospirosis IgM) and malarial infection (identified of *Plasmodium* spp. on thick or thin film). This study excluded patients with previously documented anemia (Hb less than 13 g/dl in men, 12 g/dl in women or mean corpuscular volume (MCV) value outside the range 80–100 fl), WBC count less than 5000 per cu.mm., platelet count less than 140,000 or more than 400,000 per.cu.mm, other hematologic diseases, chronic liver disease, chronic kidney disease, immunodeficiency patients, patients with malignancy during chemotherapy, and patients who were receiving any immunosuppressive drugs. We excluded the acute febrile illness patients with uncertain diagnosis. The dengue infected patients with evidence of co-infection were also excluded. The sample size was calculated using the formula *N* = *p* × (100 - P) × z2/d2 in which P was the anticipated prevalence, d was the desired precision and z was the appropriate value from the normal distribution for the desired confidence. We estimated an anticipated prevalence of 20% with 95% confidence (*Z* = 1.96) of achieving a precision of 10%. The calculated sample size was 300 patients, divided in to 2 groups; the dengue group and control group. The data concerning the control group was limited due to the lack of serological confirmed diagnosis of any one particular disease. To achieve the sample size, the control group needed to include patients with varying diseases; specifically rickettsia disease, leptospirosis, malaria, and primary bacteremia.

Clinical data was collected from medical records and compared between the dengue and control groups included demographic data, clinical presentations and all parameters from the CBC. Leukopenia was defined as a total WBC count of less than 4000 per cu.mm.; thrombocytopenia was defined as a total platelet count of less than 100,000 per.cu.mm; monocytosis was defined as a monocyte level of more than 10%; eosinophilia was defined as having an eosinophil level of more than 3%, and basophilia was defined as a basophil level of more than 2% of the total WBC. The CBC parameters were collected every time the blood test was performed related to days of fever until disease recovery at tenth day or when the blood component was transfused since post transfused CBC would not be analyzed. The frequency of blood test was depend on physician decision as individual case. Complete blood counts were performed using automated hematology analyzers, Siemens ADVIA® 2120 which are calibrated for standardization of results every 6 months.

The data was analyzed using SPSS statistical software version 17.0. Demographic data and laboratory data were presented as descriptive statistics including frequency, percentage, mean and range. The Chi-square test was used to compare categorical variables. The CBC parameters were analyzed using the linear mixed model as there were repeated measurements which were unbalanced and there were missing observations within the data for some subjects. A *p*-value of less than 0.05 was considered as statistically significant.

## Results

A total of 154 patients were enrolled onto the dengue group, the dengue being serologically confirmed and 146 patients in the control group were enrolled. The serologic result for dengue group was positive for NS1 antigen in 57.79% (89/154), dengue IgM antibody in 27.92% (43/154) and both in 14.29% (22/154). There were 46 of 154 (29.8%) dengue infected patients classified in severe dengue infection. The control group included 103 (70.5%) patients with rickettsial disease, 30 (20.5%) with primary bacteremia, 8 (5.5%) with malaria and 5 (3.4%) with leptospirosis.

Table 1 demonstrates the baseline characteristics of the dengue and control groups. The sex and mean ages were slightly different between groups. The dengue group had a lower proportion of male patients (49.4% vs. 62.3%; *p* = 0.024) and a lower mean age (27 vs. 45 years; *p* = 0.05).

**Table 1:** Baseline characteristics of dengue and control group

	Dengue group (*N* = 154)		Control group (*N* = 146)		*p*-value
	N (%)		N (%)		
Sex					0.024
• Male	76(49.4)		91(62.3)		
• Female	78(50.6)		55 (37.7)		
Mean Age (year)	27(15–67)		45 (15–83)		< 0.001
Underlying disease	4 (2.6)		11(7.5)		0.050

When compared to the control group, the dengue group had significant presentations of headache (47.4% vs. 34.2%, *p* = 0.021), loss of appetite (34.4% vs.15.8%, *p* < 0.001), nausea (33.8% vs.15.1%, *p* < 0.001) and bleeding diathesis (5.8% vs. 0%, *p* = 0.003). Chill symptoms were found more frequently in the control group (0.6% vs. 22.6%, *p* < 0.001). The others symptoms including myalgia, arthralgia, abdominal pain, rash, sore throat and diarrhea were not statistically different when the groups were compared (Table 2).

**Table 2:** Comparison of the clinical presentations in dengue and control patients

	Dengue (*N* = 154)		Control (*N* = 146)		*p*-value
	N	Percent	N	Percent	
Fever	154	100	146	100.0	
Chills	1	0.6	33	22.6	< 0.001
Headache	73	47.4	50	34.2	0.021
Myalgia	75	48.7	63	43.2	0.335
Rash	10	6.5	8	5.5	0.712
Arthralgia	2	1.3	5	3.4	0.223
Abdominal pain	9	5.8	7	4.8	0.686
Nausea	52	33.8	22	15.1	< 0.001
Bleeding	9	5.8	0	0	0.003
Loss of appetite	53	34.4	23	15.8	< 0.001
Sore throat	14	9.1	12	8.2	0.789
Diarrhea	8	5.2	15	10.3	0.098

**Fig. 1:**
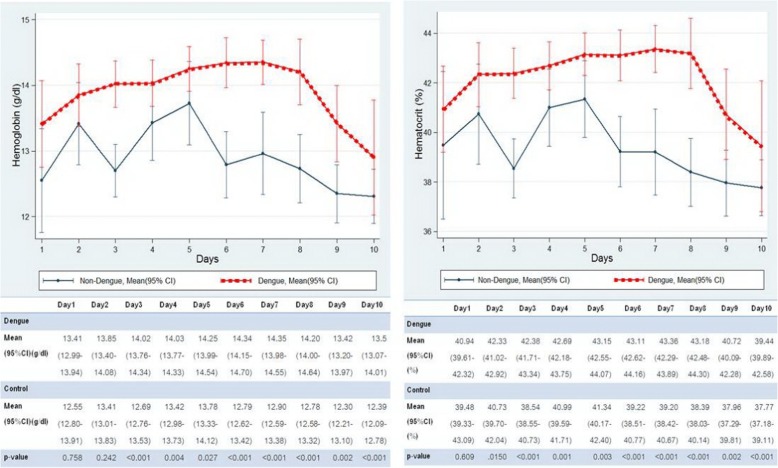
Comparison of hemoglobin and hematocrit between dengue and control group by day of fever. Shows that the dengue group had higher hemoglobin and hematocrit significantly on day 3 to day 10 (highest on day7)

There were several hematologic parameters from the CBC which were diverse in the dengue patients. Also this study identified the days of fever when these parameters were statistically significantly different between the two groups. The dengue group had significantly higher hematocrit and also higher hemoglobin levels than the control group from day 3 to day 10 (Fig. 1). The highest was on day 7 of the fever: hemoglobin level [14.3 g/dl (13.98–14.55) vs. 12.9 g/dl (12.59–13.38)] and hematocrit [43.3% (42.29–43.89) vs. 39.2% (38.42–40.67)], respectively (*p* < 0.001).

**Fig. 2:**
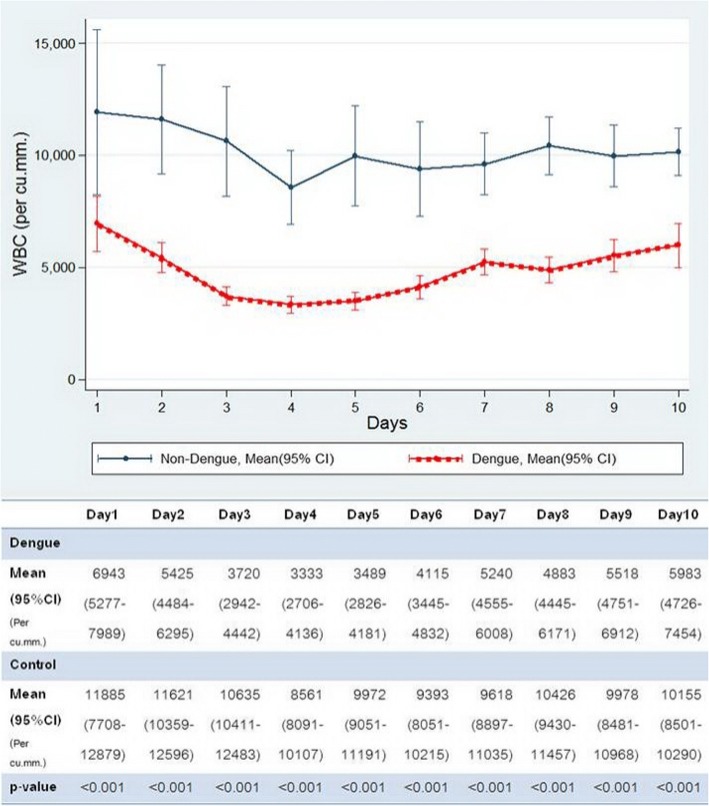
Comparison of WBC between dengue and control group by day of fever. Shows that the dengue group had lower white blood cell (WBC) count significantly from day 1 to day 10 (lowest on day 4)

As shown in Fig. 2, the dengue group had a lower total WBC count than the control group. The lowest mean WBC count was on day 4 of the fever; [3333 (2706 – 4136) vs. 8561 (8091 - 10,107) per cu.mm, *p* < 0.001]. Leukopenia was found from day 2 of fever (30.8%) and the incidence increased on successive days of the fever until day 5 (78.8%) and then there was a gradual recovery (Table 3).

**Table 3:** CBC parameters of dengue and control groups by day of fever

Day of fever	Avaliable CBC data		Leukopenia		Monocytosis		Neutrophil < 50%		Eosinophilia		Thrombocytopenia	
	Dengue (*N* = 154)	Control (*N* = 146)	Dengue N(%)	Control N(%)	Dengue N(%)	Control N(%)	Dengue N(%)	Control N(%)	Dengue N(%)	Control N(%)	Dengue N(%)	Control N(%)
**1**	16	12	0(0)	0(0)	10(62.5)	1(8.3)	0(0)	0(0)	2(12.5)	1(8.3)	0(0)	1(8.3)
**2**	39	27	12(30.8)	2(7.4)	28(71.8)	3(11.1)	3(1.9)	0(0)	1(2.6)	2(7.4)	3(7.7)	4(14.8)
**3**	61	32	40(65.6)	0(0)	31(50.8)	9(28.1)	10(16.4)	0(0)	2(3.3)	8(25)	18(29.5)	6(18.8)
**4**	69	33	53(76.8)	2(6.1)	31(44.9)	6(18.2)	18(26.1)	4(12.1)	4(5.8)	3(9.1)	36(52.2)	2(6.1)
**5**	80	29	63(78.8)	3(10.3)	33(41.3)	6(20.7)	30(37.5)	3(10.3)	5(6.3)	4(13.8)	46(57.5)	5(17.2)
**6**	75	29	44(58.7)	0(0)	36(48)	9(31)	52(69.3)	4(13.8)	16(21.3)	3(10.3)	60(80)	4(13.8)
**7**	66	30	20(30.3)	1(3.3)	26(39.4)	5(16.7)	59(89.4)	4(13.3)	9(13.6)	4(13.3)	50(75.8)	11(36.7)
**8**	42	32	12(28.6)	1(3.1)	13(31)	6(18.8)	35(83.3)	3(9.4)	7(16.7)	5(15.6)	29(69)	11(34.4)
**9**	25	20	4(16)	0(0)	9(36)	9(45)	20(80)	7(35)	7(28)	0(0)	9(36)	6(30)
**10**	15	45	3(20)	0(0)	11(73.1)	14(31.1)	11(73.3)	11(24.4)	3(20)	3(6.7)	4(26.7)	3(6.7)

**Fig. 3:**
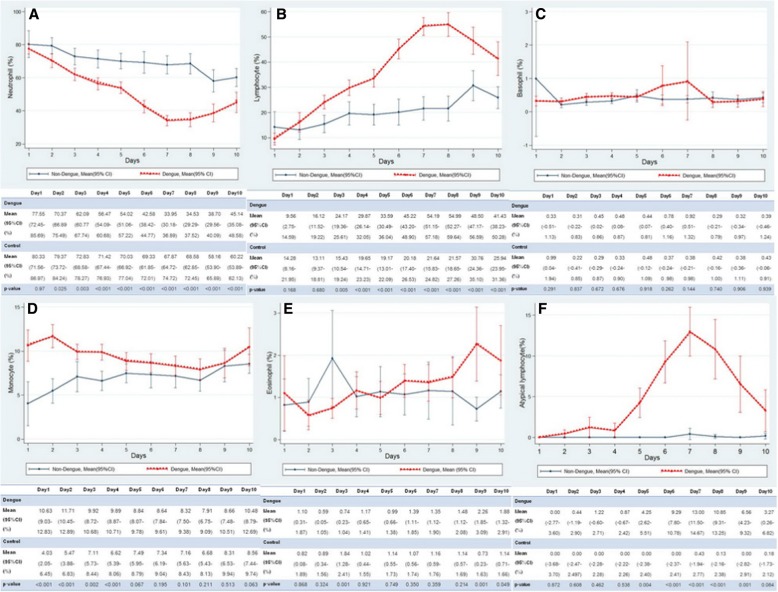
Comparison of differential WBC count between dengue and control group by day of fever. Shows that the dengue group had lower neutrophil on day 4–10 (**a**), higher lymphocyte on day 3–10 (**b**), basophils not different (**c**), higher monocytes on day 1–4 (**d**), higher eosinophils on day 9–10(**e**) and higher atypical lymphocytes on day 5–9 (**f**)

**Fig. 4:**
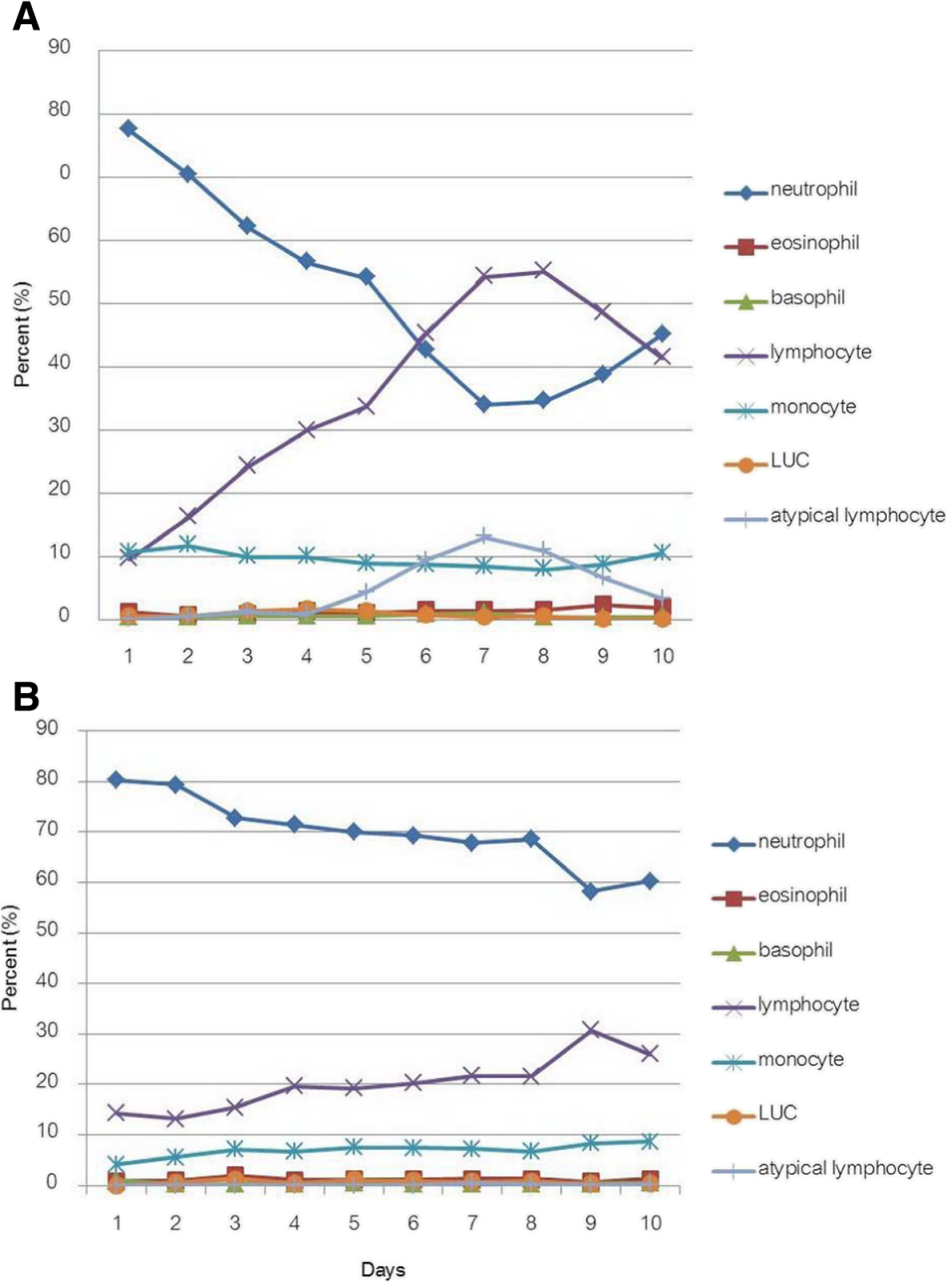
The change in differential WBC count of dengue and control group by day of fever. Shows differential white blood cell count of dengue group (**a**) and control group (**b**). The neutrophil to lymphocyte ratio of dengue group was > 1 on the first 5 days then reversed on day 6 to Day 9 but in control group, the ratio was always > 1

The differential WBC count between the dengue and controls group by day of the fever are demonstrated in Fig. 3. The dengue group had a higher monocyte percentage than the control group on day 1 to 4 with the highest being on day 2 [11.7 vs. 5.4% (*p* < 0.001)]. Monocytosis was found in 62.5, 71.8, 50.8 and 44.9% of patients on days 1 to 4 of the fever respectively. The neutrophil percentage of the dengue group gradually decreased in a negative correlation with the increase in the percentage of lymphocytes in successive days of the fever. Dengue patients had higher neutrophil percentage predominately in the first 5 days of the fever then this was reversed and the percentage of lymphocytes increased. Reversed neutrophil to lymphocyte ratios occurred on day 6 to 9 of fever (69.3, 89.4, 83.3 and 80% respectively). On the other hand, the neutrophil percentage in the control group predominated from the first day of the fever until recovery (Fig. 4). The dengue group had significantly higher percentage of atypical lymphocytes on day 5 to 9 of the fever than did the control group [4.25 vs. 0%; *p* = 0.004, 9.29 vs.0%; *p* < 0.001, 13 vs. 0.48%; *p* < 0.001, 10.85 vs. 0.13%; *p* < 0.001 and 6.56 vs. 0%; *p* = 0.001 respectively] with the highest being on day 7. The mean eosinophil percentage was higher in cases of dengue infection on day 9 and 10 (2.2 vs. 0.7% and 1.88 vs. 1.14%, *p* < 0.001). Eosinophilia was found in 28% and 20% of dengue patients on day 9 and 10 respectively. Basophil percentages were no different between the groups.

**Fig. 5:**
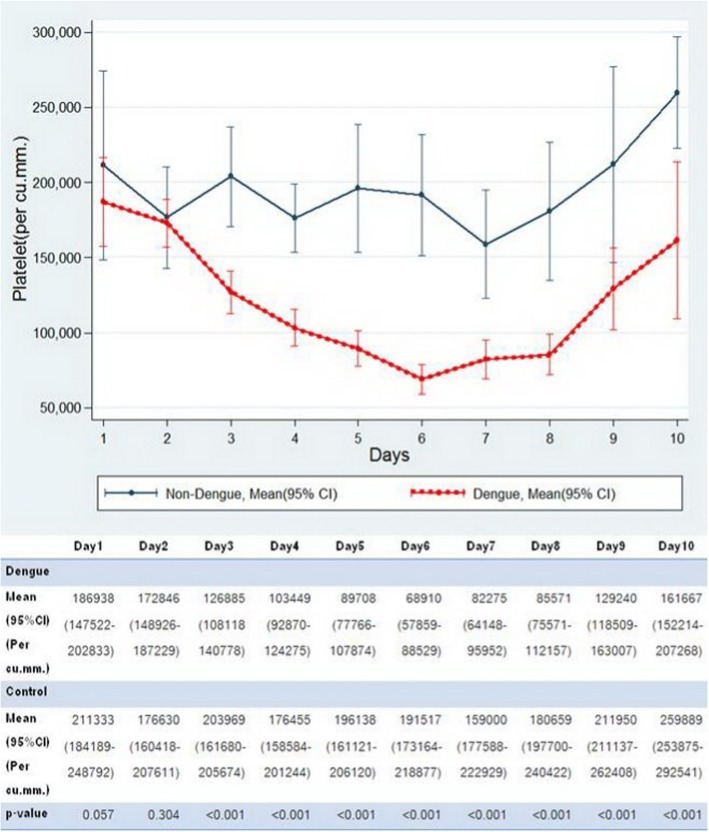
Comparison of platelet count between dengue and control group by day of fever. Shows that the dengue group had significant lower platelet count from day 3 to day 10 (lowest on day 6)

The mean platelet count was lower in the dengue than the control group on day 3 to 10 with the lowest on day 6 (68,910 vs. 196,137 per cu.mm., *p* < 0.001) as shown in Fig. 5. The thrombocytopenia occurred in more than 50% of patients on day 4 and reached the highest level of 80% on day 6.

## Discussion

Dengue infected patients present with acute febrile illness without localized signs and symptoms and the clinical presentations may resemble other infections hence making a differential diagnosis difficult in distinguishing it from other infections such as tropical infection (rickettsial disease, leptospirosis or malaria), other viral infection and primary bacteremia. Our study identified the significant differences of clinical features and CBC parameters to facilitate the distinguishing of dengue infection from the other causes.

The clinical presentations in dengue infection are fever, headache, loss of appetite, nausea, bleeding diathesis, myalgia, abdominal pain, sore throat and diarrhea [5, 8, 9]. These symptoms are not specific and can be found in other infections. From our study, we found that headache, nausea, loss of appetite and bleeding diathesis were commonly found in dengue patients but chills presented significantly more frequently in the control group. Therefore, these clinical presentations may be helpful in distinguishing dengue infection from the other infections at presentation.

The demographic data between dengue and the control group in our study were different as regards gender, mean age and preexisting underlying diseases. The mean age of the dengue group was 27 years. They showed a strong correlation with the epidemiology of known dengue infection in Thailand, namely that the highest proportion of cases by age group is 15–24 years [2]. The control group was older (mean age of 45 years old) which may be explained by the higher level of preexisting underlying diseases in this group compared to the dengue group.

In addition to the clinical features, there were several hematologic parameters that were useful in distinguishing dengue infection from other infections using the CBC which is an accessible laboratory test in almost all hospitals.

In summary, the dengue group had higher hemoglobin levels and a higher hematocrit from day 3 to day 10 (highest on day 7), lower white blood cell (WBC) count from day 2 to day 10 (lowest on day 4) and lower platelet count from day 3 to day 10 (lowest on day 6). The details of the differential WBC percentage were that the samples from the patients with dengue showed higher monocyte on day 1–4 (highest on day 2), higher atypical lymphocytes day 5–9 (highest on day 7) and higher eosinophils on day 9–10 (highest on day 9) than control group. Furthermore, the neutrophil to lymphocyte ratio in the dengue group was > 1 on the first 5 days then reversed on day 6 to day 9.

The dengue group had higher hemoglobin levels and a higher hematocrit as a result of the plasma leakage. An in vitro study revealed a cross-reaction of proinflammatory mediators such as tumor necrosis factor (TNF)-alpha and anti-NS1 antibodies with surface proteins on endothelial cells causing apoptosis of these cells and subsequently plasma leakage [10].

The total white blood cell count was significantly lower in the dengue group than the control group. Leukopenia occurred from day 2 and was lowest on day 5 of the fever in the dengue group. A hypothesis regarding the occurrence of the leukopenia in the cases of dengue infection was that it was caused by the destruction or inhibition of myeloid progenitor cells as the bone marrow examination showed mild hypocellularity in the first seven days of fever then normal cellularity in the convalescent phase [11].

Monocytosis occurred in 60–70% of patients in our study which was similar to previous study which found it in 84.6% of patients [12]. Another study showed that monocytosis was found in cases of dengue hemorrhagic fever more often than dengue fever [8]. Thus, monocytosis might be a parameter which can be used to predict the severity of dengue infection. A hypothesis as to why there is an increase in monocytes in the first few day of the fever is that monocytes and macrophages are the part of the primary immune which carry out phagocytosis of microorganisms and present the resulting carried antigen to the T helper cells. However, there were several conditions associated with monocytosis, for example other viral infections, enteric fever, malaria, tuberculosis, HIV, malignancy or pyrexia of unknown origin [13], so the monocytosis was not specific to dengue infection.

The neutrophils percentage was predominant in the first 5 days of the fever, a condition which was reversed, lymphocytes then predominating. This result was in agreement with a previous study which showed that lymphocytes predominated on day 10 of the fever [14].

A study from Brazil and Pakistan had similar results to this study in terms of eosinophilia, the study showing that around 20% of patients had a higher eosinophil count on day 10 of the fever [5, 9]. In cases of dengue infection, eosinophil levels were low in the acute phase due to the response to the inflammatory process, the levels then returning to baseline and increasing in the convalescence phase [15].

Atypical lymphocytes increased on days 5–9 in the dengue group. The higher atypical lymphocyte percentage was found in cases of dengue hemorrhagic fever more than dengue fever [12]. Therefore the percentage of atypical lymphocytes may be another parameter useful in the prediction of the severity of dengue infection in addition to monocytosis.The study from India showed basophilia (basophil > 2%) in 52.9% of dengue patients [12]. On the contrary to this result, the basophils were not elevated in our study. The cause of basophilia may be due to recovery from the bone marrow suppression in the convalescence phase [16].

Half of the patients had thrombocytopenia on day 4 and increased up to approximately 80% of cases on day 6. Although almost patients had thrombocytopenia, but most of them were non-severe form of dengue infection so the bleeding diathesis of our study was low(5.8%). There are several hypotheses to explain this such as an infected megakaryocyte by the virus, peripheral destruction and cross-reaction of antibodies against platelets [17]. The platelets of dengue infected patients had mitochondrial dysfunction which activated the apoptosis cascade and led to cell death [18]. Prolonged thrombocytopenia was found in dengue hemorrhagic fever more frequently than in cases of dengue fever [12], so the duration of thrombocytopenia is considered to be the predictor of the severity of dengue infection. Currently, the new parameter to reflect the rate of thrombopoiesis is the immature platelet fraction (IPF) which can be used to predict platelet recovery in dengue patients [19]. Cytopenia is the major parameter from the CBC which can distinguish dengue infection from the others. A review of a bone marrow study in dengue patients showed the transient suppression of hematopoiesis within 3–4 days of infection then the host inflammatory response which occurred to eliminate infected cells. Therefore, the cytopenia is probably a protective mechanism to limit injury to the marrow stem cells during the subsequent process of the eradication of infected cells [16]. In addition, the dengue-infected endothelial cells are potentially bound to white blood cells, neutrophils, lymphocytes, platelets, and large lymphocytes in vitro but the monocytes, basophils, and eosinophils had no interaction. The increased binding of neutrophils and platelets to infected endothelial cells may explain neutropenia and thrombocytopenia in dengue patients [20].

In addition to a previous study, our study included the changes in all CBC parameters on each successive day of the fever. The first parameter was monocytosis, followed by leukopenia, thrombocytopenia, a raised hematocrit, increased atypical lymphocytes and a reversed neutrophil to lymphocyte ratio respectively. The recovery phase started with the increase of white blood cell, hematocrit and platelet. We also found eosinophilia at this phase.

A study in Indonesia identified the possible use of new parameters to enable the differentiation of dengue from leptospirosis and enteric fever using flow cytometry to quantitate the atypical lymphocyte area, high-fluorescent lymphocyte counts, immature granulocytes and IPF [21]. These investigations may give high accuracy for diagnosis but are inaccessible in all hospitals. Furthermore, there were other parameters which would probably be useful for diagnosis of dengue infection, such as prolonged activated partial thromboplastin time, prothrombin time, thrombin time and elevated liver enzymes [22], but these data in our cohort were limited. Many laboratory parameters are not only useful for diagnosis but also enable the prediction of severity such as monocytosis, duration of thrombocytopenia, high atypical lymphocyte percentage, high serum lactate and lactate dehydrogenase levels [23, 24]. The last enzyme test was not performed routinely in our study.

The limitation of this study was that it was a retrospective study. Much information which may be useful for comparison and the diagnosis of dengue infection was not available from the database and as it was retrospective could not be addressed. The control group was made up of patients with many diseases which make the data inhomogeneous when comparing it with the dengue group especially malaria or leptospirosis which were few cases in that group so some data may be masked. Hence, other CBC parameters and further work needs to be done using a specific disease group to compare to the dengue group. Another limitation, the CBC result was done on physician decision as individual case. This limitation might influence the results obtained in our study as the CBC was not done on the daily basis. The enrollment in the study was done from diagnosis and reviewed for history of fever which was matched the inclusion criteria. This may effect the selection bias of case and quality of the study. Prospective study in this topic can be done in the future to correct the limitation of our study.

## Conclusions

We identified important clinical presentations and useful CBC parameters to enable the differentiation of dengue patients from other patients with other causes of acute febrile illness. These findings can be applied to local hospital situations as the data can be amassed from a CBC. An accurate diagnosis using these data will enable further investigation to be tailored and early treatment for the patient.

## Abbreviations

CBC: Complete blood count
Hb: Hemoglobin
Hct: Hematocrit
IPF: Immature platelet fraction
MCV: Mean corpuscular volume
TNF: Tumor necrosis factor
WBC: White blood cell count
